# Agricultural Soils Amended With Thermally-Dried Anaerobically-Digested Sewage Sludge Showed Increased Risk of Antibiotic Resistance Dissemination

**DOI:** 10.3389/fmicb.2021.666854

**Published:** 2021-04-28

**Authors:** Leire Jauregi, Lur Epelde, Itziar Alkorta, Carlos Garbisu

**Affiliations:** ^1^Department of Conservation of Natural Resources, NEIKER—Basque Institute for Agricultural Research and Development, Basque Research and Technology Alliance (BRTA), Derio, Spain; ^2^Department of Biochemistry and Molecular Biology, University of the Basque Country (UPV/EHU), Bilbao, Spain

**Keywords:** antibiotic resistance genes, emerging contaminants, mobile genetic elements, organic fertilization, soil microbial diversity, soil quality

## Abstract

The application of sewage sludge (SS) to agricultural soil can help meet crop nutrient requirements and enhance soil properties, while reusing an organic by-product. However, SS can be a source of antibiotic resistance genes (ARGs) and mobile genetic elements (MGEs), resulting in an increased risk of antibiotic resistance dissemination. We studied the effect of the application of thermally-dried anaerobically-digested SS on (i) soil physicochemical and microbial properties, and (ii) the relative abundance of 85 ARGs and 10 MGE-genes in soil. Soil samples were taken from a variety of SS-amended agricultural fields differing in three factors: dose of application, dosage of application, and elapsed time after the last application. The relative abundance of both ARGs and MGE-genes was higher in SS-amended soils, compared to non-amended soils, particularly in those with a more recent SS application. Some physicochemical parameters (i.e., cation exchange capacity, copper concentration, phosphorus content) were positively correlated with the relative abundance of ARGs and MGE-genes. Sewage sludge application was the key factor to explain the distribution pattern of ARGs and MGE-genes. The 30 most abundant families within the soil prokaryotic community accounted for 66% of the total variation of ARG and MGE-gene relative abundances. Soil prokaryotic α-diversity was negatively correlated with the relative abundance of ARGs and MGE-genes. We concluded that agricultural soils amended with thermally-dried anaerobically-digested sewage sludge showed increased risk of antibiotic resistance dissemination.

## Introduction

In the current scenario of increasing world population and environmental degradation, the transition to a Circular Economy model requires, among many other aspects, the reuse and sustainable management of wastes and by-products. The application of sewage sludge (SS) to soil is a common agricultural practice that can certainly lead to agronomic improvements such as, for instance: (i) increased soil organic matter (OM) and nutrient content (Lashermes et al., 2009); (ii) enhanced soil porosity and bulk density (Singh and Agrawal, 2007; Annabi et al., 2011); (iii) greater water holding capacity (Bulluck et al., 2002); (iv) higher soil microbial activity (García et al., 1992); and (v) improved overall soil quality (Powlson et al., 2011). The application of organic amendments (e.g., SS) into soil can also result in structural and functional changes in soil bacterial communities (Innerebner et al., 2006; Epelde et al., 2014).

However, the application of SS to agricultural soil as organic amendment can likewise lead to potential risks for human and environmental health, in particular, owing to the presence in SS of: (i) elevated concentrations of potentially toxic metals (Singh and Agrawal, 2007); (ii) organic contaminants (Alvarenga et al., 2015); (iii) nanoparticles (Fijalkowski et al., 2017); (iv) microplastics; and (v) pharmaceutical compounds (Martín et al., 2015), including antibiotics and their transformation products. These later chemical emerging contaminants (antibiotics and their transformation products) are often accompanied in SS by several biological contaminants of great concern, i.e., antibiotic resistance genes (ARGs), antibiotic resistant bacteria (ARB) and mobile genetic elements (MGEs).

Most worryingly, the use, overuse and misuse of antibiotics for medical and veterinary use have promoted the emergence and spread of antibiotic resistance in the environment, including the soil ecosystem (Cytryn, 2013). Antibiotic resistant bacteria can transfer to other bacteria (including potential human pathogens) the ARGs they harbor through horizontal gene transfer (HGT) via a variety of MGEs such as plasmids, transposons, integrative conjugative elements, phages, integrons, genomic islands, etc. (Pruden et al., 2006; Zhu et al., 2013).

In the European Union, in 2014, the consumption of antimicrobials for medical and veterinary use, including animal production, reached a staggering value of 12,720 tons of active substance (European Centre for Disease Prevention and Control [ECDC], European Food Safety Authority [EFSA], and European Medicines Agency [EMA], 2017). It has been reported (Van Boeckel et al., 2017) that, worldwide, more than 73% of all antimicrobials are administered to animals for veterinary or food-producing purposes. Relevantly, a considerable amount (between 30 and 90%) of the antibiotics administered for human or veterinary purposes are excreted in the urine and feces, essentially unchanged or as active metabolites (Sarmah et al., 2006). Regrettably, wastewater treatment plants (WWTPs) are not designed to efficiently remove these emerging contaminants and, then, they are unsurprisingly regarded as hotspot for the emergence and dissemination of antibiotic resistance (Rizzo et al., 2013).

In SS, the concentration of widely use antibiotics ranges from μg kg^–1^ to mg kg^–1^ (Lillenberg et al., 2009; Nieto et al., 2010). Although there are certainly many differences between countries and specific WWTPs, resulting in the impossibility to describe a common pattern, several authors (Zhang and Li, 2011; Rutgersson et al., 2020) detected lower levels of sulfonamides, macrolides and tetracyclines, compared to quinolones, in SS samples.

In the European Union, the most common method of SS disposal remains its application to agricultural soil, followed by thermal disposal and landfill (Christodoulou and Stamatelatou, 2016). The improper disposal of SS can lead to serious environmental risks such as, for instance, the contamination of aquifers and soils with a variety of potentially toxic inorganic and organic compounds (Sharma et al., 2017). The majority of legislations on SS management, such as Directive 86/278/ECC dealing with soil protection when SS is used in agriculture (European Commision, 1986), do not consider the abovementioned emerging contaminants. Actually, these legislations have traditionally been focused on total metal concentrations, both in the SS itself and in the amended agricultural soil, as well as on the presence of potential human pathogens.

The aim of this study was to assess the impact of the application of thermally-dried anaerobically-digested SS on: (i) soil physicochemical and microbial properties, including soil prokaryotic diversity and composition; and (ii) the presence and relative abundance of ARGs and MGE-genes in soil. In particular, regarding the application of SS, three factors were studied: dose of application, dosage of application, and elapsed time after the last application. The term “dose” refers here to a specific amount of SS applied at one time. Instead, the term “dosage” refers to the total amount of SS applied to those fields (i.e., the sum of all the individual applications). The novelty of the study is supported by the following facts: (1) the study was carried out with samples taken from a high number of real agricultural farms, i.e., 20 farms; (2) the studied agricultural fields differed in the abovementioned three factors, thus allowing the assessment of their individual and combined influence on the main topic under study, i.e., antibiotic resistance; and (3) the SS had been thermally-dried and anaerobically-digested prior to their application which, *a priori*, could have considerably reduced the risk of antibiotic resistance spread. We hypothesized that the application of SS would enhance soil physicochemical and microbial properties, alter the composition of soil prokaryotic communities and, finally, increase the relative abundance of ARGs and MGE-genes in soil. Moreover, we expected these changes to be dependent upon the three factors mentioned above. This study was carried out with SS from a single WWTP. Nonetheless, SS composition and management (e.g., storage, treatment, dose, and mode of application, etc.) can significantly differ between WWTPs. This fact must be taken into consideration when comparing our results with those from other studies.

## Materials and Methods

### Experimental Design

The study was carried out with soil samples collected from 20 real agricultural fields (ranging from ca. 1 to 14 ha) located in the Valley of Orba (Valdorba), province of Navarre (North of Spain). The Valley of Orba is composed of a series of valleys occupying a total area of 130 ha (46% of that area corresponds to agricultural land). Thermally-dried anaerobically-digested SS from a local WWTP had been applied to 13 out of those abovementioned 20 agricultural fields. The physicochemical properties of the SS were: pH = 8.1; dry matter content = 17.3%; C/N ratio = 5.6; total metal concentrations < 3, 73, 196, 40, 47, and 936 mg kg^–1^ dry weight-DW SS for Cd, Cr, Cu, Ni, Pb, and Zn, respectively.

Regarding the application of SS, these 13 agricultural fields differed in the following three factors: (i) *dose of application* = 22, 33 and 44 t ha^–1^; (ii) *dosage of application* = 22, 55, 77, and 99 t ha^–1^; and (iii) *elapsed time after the last application* = 1, 2, 3, and 4 years ago. Seven fields had never been amended with SS and were then used as control unamended soils.

As described above, soil samples were collected from each agricultural field to study the impact of the application of SS on soil physicochemical and microbial properties, as well as on the presence and relative abundance of ARGs and MGE-genes in soil. In particular, three composite soil samples, each composed of 10 soil cores (depth: 0–10 cm) randomly taken in an area of 10 m^2^, were collected per agricultural field, each of them corresponding to a different area within such field. The three areas were at least 50 m apart from each other. At soil sampling times, the agricultural fields were planted with different crops (barley, beans, rapeseed, wheat). Soil samples were collected in polyethylene bags, protected from sunlight, and immediately transferred to the laboratory. The experimental design is summarized in Table 1.

**TABLE 1:** Experimental design: description of the 20 agricultural fields.

Field	SS applications (year)	Elapsed time after the last application (years)	Dose (t ha^–1^)	Dosage (t ha^–1^)	Current crop
1	2009 + 2013	4	33/22	55	Rapeseed
2	−		−	−	Barley
3	2007 + 2011 + 2015	2	33/22/22	77	Barley
4	−		−	−	Wheat
5	2006 + 2010 + 2014	3	44/33/22	99	Wheat
6	−		−	−	Wheat
7	2008 + 2012 + 2016	1	33/22/22	77	Wheat
8	−		−	−	Wheat
9	2006 + 2010 + 2014	3	44/33/22	99	Wheat
10	2013	4	22	22	Barley
11	−		−	−	Beans
12	2008 + 2012 + 2016	1	33/22/22	77	Barley
13	2007 + 2011 + 2015	2	33/22/22	77	Barley
14	2009 + 2013	4	33/22	55	Rapeseed
15	−		−	−	Barley
16	2006 + 2010 + 2014	3	44/33/22	99	Wheat
17	2008 + 2012 + 2016	1	33/22/22	77	Wheat
18	−		−	−	Wheat
19	2005 + 2009 + 2013	4	44/33/22	99	Wheat
20	2007 + 2011 + 2015	2	33/22/22	77	Rapeseed

### Soil Physicochemical Properties

Prior to the determination of soil physicochemical parameters, soil samples were air-dried at room temperature until constant weight. The following parameters were determined in SS and soil samples according to standard methods (MAPA, 1994): OM content, pH, cation exchange capacity (CEC), electrical conductivity (EC), texture, water soluble organic carbon (WSOC), total nitrogen (N), Olsen phosphorus (P), and content of nitrate (NO_3_^–^), ammonium (NH_4_^+^), and potassium (K^+^). Inductively coupled plasma-optical emission spectrometry (ICP-OES) was used for the determination of pseudo-total metal concentrations following aqua regia digestion (McGrath and Cunliffe, 1985). CaCl_2_-extractable (0.01 M), NaNO_3_ extractable (0.1 M), and low molecular weight organic acid (LMWOA) solution-extractable metal fractions in soil and SS were determined following Gupta and Aten (1993); Houba et al. (2000), and Feng et al. (2005), respectively.

### Soil Microbial Properties

For the determination of soil microbial parameters, fresh soil samples were sieved to < 2 mm and stored at 4°C for less than a month prior to their analysis. Microbial biomass carbon was determined according to Vance et al. (1987). Soil respiration was measured following ISO 16072 (2002). For the molecular analyses, DNA was extracted from soil samples (0.25 g DW soil) using the Power Soil^TM^ DNA Isolation Kit (MoBio Laboratories Inc., Carlsbad, CA). Prior to DNA extraction, soil samples were washed twice in 120 mM K_2_PO_4_ (pH 8.0) to wash away extracellular DNA (Kowalchuk et al., 1997). DNA concentration was determined using a NanoDrop spectrophotometer (ND-1000, Thermo Fisher Scientific, Wilmington, DE). The extracted DNA was stored at −20°C until use.

Amplicon libraries preparation was carried out as described in Lanzén et al. (2016), using a dual indexed adapter with sequence-specific primers for prokaryotic communities, targeting the V4 region of the 16S rRNA genes. Primers were 519F (CAGCMGCCGCGGTAA) adapted from Ovreås et al. (1997) and 806R (GGACTACHVGGGTWTCTAAT) from Caporaso et al. (2012). Sequencing was carried out with an Illumina MiSeq V2 platform and paired-end sequencing strategy (2 × 250 nt) at Tecnalia, Spain. Reads were merged, quality filtered and clustered into operational taxonomic units (OTUs) as described in Lanzén et al. (2016). The taxonomic classification was performed using CREST (Lanzén et al., 2012).

High-throughput real-time PCR (HT-qPCR) was employed to quantify the abundance of ARGs and MGE-genes using the nanofluidic qPCRBioMarkTM HD system with 48.48 and 96.96 Dynamic Array Integrated Fluidic Circuits (IFCs) (Fluidigm Corporation), following Urra et al. (2019a). A total of 96 validated primer sets (Hu et al., 2016) were used: 85 primer sets targeting ARGs conferring resistance against all major classes of antibiotics [10 aminoglycosides, 14 β-lactams, 5 FCA (fluoroquinolone, quinolone, florfenicol, chloramphenicol and amphenicol), 13 MLSB (macrolide, lincosamide, streptogramin B), 5 multidrugs, 4 sulfonamides, 24 tetracyclines and 10 vancomycines], 10 primer sets targeting MGE-genes (8 genes encoding transposases, 2 genes encoding integrases) and one primer set for the 16 rRNA gene. Measurements were conducted in the Gene Expression Unit of the Genomics Facility of SGIker—University of the Basque Country, Spain. Raw data were processed with the Fluidigm Real-Time PCR Analysis Software (v.3.1.3) with linear baseline correction and manual threshold settings. A threshold cycle (C_T_ value) of 31 was chosen, as the highest C_T_ value obtained in our study was 30.5. The detection of the ARGs and MGE-genes was considered positive when at least 3 of the 4 technical replicates for each sample were above the detection limit. The relative copy number was calculated as the proportion of the abundance of a given ARG or MGE-gene to the abundance of the 16S rRNA gene (Looft et al., 2012).

### Statistical Analysis

The calculation of α-diversity indices for the studied soil prokaryotic communities, as well as the treatment of 16S rRNA gene amplicon sequencing data, were performed with R package *vegan* (Oksanen et al., 2015). Rarefied richness was calculated to compensate for the observed variation in read numbers across samples (it was estimated by means of interpolating the expected richness at the lowest sample-specific sequencing depth). Differences in the abundance of prokaryotic taxa at family level among plots were determined, followed by Bonferroni’s multiple comparisons test, using R software (v.3.5.1).

The statistical significance of the observed differences in the values of soil physicochemical properties, microbial properties and ARG and MGE-gene relative abundances between SS-amended and unamended soils, and ARG and MGE-gene relative abundances in our SS, were determined by Welch’s *t*-test (for unequal variances and unequal sample sizes) using package *agricolae* of R software (v.3.5.1). All statistical tests were considered significant at *p* < 0.05, except for Bonferroni correction.

Relationships between (i) SS experimental factors (presence/absence of SS, dose of application, dosage of application, elapsed time after the last application); (ii) soil physicochemical properties; (iii) abundance of ARGs and MGE-genes; (iv) the most abundant prokaryotic families; and (v) values of α-diversity indices were explored by redundancy analysis (RDA) using Canoco 5 (ter Braak and Smilauer, 2012). Response data were log transformed and centered, and the number of permutations was unrestricted. Redundancy analyses were performed after forward selection in which only those explanatory variables that contributed significantly to the analysis were taken into account. In order to study the influence of SS application on the relative abundance of ARGs and MGE-genes, the abovementioned SS experimental factors were used as explanatory variables. In the same way, RDAs were conducted to find out how much of the variability in the relative abundance of ARGs and MGE-genes could be attributed to the: (i) soil physicochemical properties; (ii) the α-diversity of soil prokaryotic communities; and/or (iii) the composition of soil prokaryotic communities (i.e., the 30 most abundant families). The variables *field* and *current crop* were used as covariates. Kendall’s rank correlation coefficients (followed by Bonferroni’s multiple comparisons test) between prokaryotic taxa at family level and the relative abundance of ARGs and MGE-genes were obtained using R software (v.3.5.1). A network analysis was performed to explore the correlations between multi-resistant prokaryotic families and ARGs and MGE-genes. Network visualization was conducted in Gephi platform.

A principal component analysis (PCA) was performed to reduce the dimensionality of the soil physicochemical properties (i.e., dry matter, OM, WSOC, pH, CEC, EC, NO_3_^–^, NH_4_^+^, total N, Olsen P, K^+^, clay, and loam content). Two linear axes, which explained the maximum amount of variance, were selected for Structural Equation Modelling (SEM) analysis (Curiel Yuste et al., 2019). The first axis (PC1, 28.8% of the explained variance) was positively correlated with total N, K^+^, Olsen P, OM, clay content, and CEC. By contrast, PC1 was negatively correlated with WSOC, NH_4_^+^, and pH. The second axis (PC2, 17.7% of the explained variance) was positively correlated with NO_3_^–^, EC and loam content. The heavy metal (HM) pollution index was calculated as the mean of the ratios between the concentration of each specific HM and its corresponding regulatory limit according to “Law 4/2015, on prevention and correction of soil contamination in the Basque Country (BOE-A-2015-8272).” Structural equation models were used to assess the direct and indirect influence of biotic (prokaryotic diversity indices) and abiotic (dosage of application, elapsed time after the last application, soil physicochemical parameters, HM pollution index) factors on ARGs and MGE-genes. The variables *field* and *current crop* were considered as random factors in the SEM, which was performed using *piecewiseSEM* package in R (Lefcheck, 2016). The Shipley’s direct separation test was used to assess the overall fit of the models. Models were accepted when Fisher’s C statistic was above the significance level (*p* < 0.05). The Akaike’s Information Criterion (AIC) was used to perform accepted model comparisons. Standardized path coefficients, which describe the strength and sign of the relationship between two variables, were estimated by using the maximum likelihood algorithm (Shipley, 2018).

## Results

### Physicochemical and Microbial Properties of SS-Amended and Unamended Soils

Soil physicochemical properties varied considerably among the 20 studied fields (Table 2). Soils had a loam or clay loam texture and showed the following physicochemical properties: OM content = 2.28%, WSOC = 99 mg kg^–1^, pH = 8.4, CEC = 14.4 mEq 100 g^–1^, EC = 0.153 mS cm^–1^, NO_3_^–^ content = 101.4 mg kg^–1^ and NH_4_^+^ content = 2.24 mg kg^–1^ (Table 2). Values of extractable metals were below the quantification limit. The application of SS significantly increased Olsen P and Zn content in SS-amended soils compared to unamended soils.

**TABLE 2:** Physicochemical and microbial properties of the 20 agricultural soils.

Property	Mean ± SD	Range	SS amended	Unamended
Dry matter (%)	86.5 ± 1.9	81.2–87.9	86.4 ± 2.2	86.6 ± 1.1
OM (%)	2.3 ± 0.4	1.8–3.0	2.3 ± 0.4	2.2 ± 0.4
WSOC (mg C kg^–1^ DW soil)	99.0 ± 35.5	54.2–154.4	96.4 ± 36.4	104 ± 34
Ph	8.4 ± 0.1	8.3–8.5	8.4 ± 0.1	8.4 ± 0.1
CEC (mEq 100 g^–1^)	14.4 ± 3.1	10.4–19.4	14.9 ± 3.1	13.5 ± 2.8
EC (mS cm^–1^)	0.15 ± 0.02	0.13–0.18	0.15 ± 0.02	0.15 ± 0.02
NO_3_^–^ (mg kg^–1^)	101 ± 69	41–226	103 ± 68	98.8 ± 72
NH_4_^+^ (mg kg^–1^)	2.2 ± 1.1	1.1–4.3	2.2 ± 0.9	2.4 ± 1.4
N (%)	0.16 ± 0.03	0.12–0.20	0.16 ± 0.03	0.15 ± 0.03
P (mg kg^–1^)*	25.0 ± 14.6	9.4–47.8	28.0 ± 14.6	19.4 ± 13.2
K (mg kg^–1^)	1,372 ± 621	873–2,854	1,383 ± 578	1,353 ± 708
Clay (%)	30.3 ± 4.8	25.8–36.2	30.3 ± 4.6	30.1 ± 5.2
Sand (%)	32.0 ± 5.2	25.6–39.7	32.0 ± 5.5	32.0 ± 5.0
Silt (%)	37.8 ± 3.6	34.5–41.5	37.7 ± 3.5	37.9 ± 3.8
Cd (mg kg^–1^ DW soil)	1.7 ± 0.50	1.1–2.7	1.7 ± 0.49	1.6 ± 0.53
Cr (mg kg^–1^ DW soil)	20.8 ± 5.9	13.3–29.8	21.4 ± 5.5	19.7 ± 6.5
Cu (mg kg^–1^ DW soil)	21.0 ± 5.9	13.9–30.6	22.0 ± 5.4	19.3 ± 6.4
Ni (mg kg^–1^ DW soil)	25.7 ± 5.1	18.8–33.2	26.5 ± 4.8	24.3 ± 5.3
Pb (mg kg^–1^ DW soil)	19.5 ± 5.4	12.8–26.4	20.2 ± 5.3	18.2 ± 5.3
Zn (mg kg^–1^ DW soil)**	59.3 ± 10.0	42.8–73.6	62.3 ± 8.5	53.9 ± 10.3
BR (mg C kg^–1^ DW soil h^–1^)	1.28 ± 0.21	0.98–1.56	1.28 ± 0.21	1.30 ± 0.19
MBC (mg C kg^–1^ DW soil)	636 ± 104	455–812	645 ± 103	618 ± 107
Richness (R)**	3,536 ± 622	2,670–4,390	3,390 ± 638	3,802 ± 504
Shannon’s index (H’)*	7.0 ± 0.27	6.5–7.4	6.9 ± 0.28	7.1 ± 0.23
Simpson’s index (D)*	0.997 ± 0.001	0.996–0.998	0.997 ± 0.001	0.998 ± 0.001
Pielou’s evenness (J’)	0.813 ± 0.020	0.782–0.839	0.811 ± 0.021	0.817 ± 0.020

*p < 0.05; **p < 0.01, based on Welch’s t-test.

Regarding soil microbial properties (Table 2), basal respiration (BR) values ranged from 0.98 (Field 1) to 1.56 (Field 17) mg C kg^–1^ DW soil h^–1^. In turn, values of microbial biomass carbon (MBC) ranged from 455.1 (Field 11) to 812.4 (Field 14) mg C kg^–1^ DW soil. Similarly, values of prokaryotic α-diversity ranged between 2,670 and 4,390 for richness (R); 6.51 and 7.35 for the Shannon’s index (H’); 0.996 and 0.998 for the Simpson’s index (D); and 0.782 and 0.839 for Pielou’s evenness (J’). Statistically significant (*p* < 0.001) differences between agricultural fields were observed for all α-diversity parameters. Field 9 showed the lowest values for all the α-diversity indices determined here. By contrast, Field 7 showed the highest R, H’, and D values (the highest J’ value was observed in Field 6). In addition, significantly lower R, H’, and D values were found in SS-amended vs. unamended soils.

Regarding soil prokaryotic community composition, 79.1, 67.3, and 34.5% of the 16S rRNA gene amplicon reads were taxonomically classified to order, family and genus rank, respectively. Out of the 2500 classified families, statistically significant differences in family abundance among agricultural soils were found for only 13 families. Three of these 13 families were included among the 30 most abundant families detected in our study: *Cytophagaceae*, *Methylobacteriaceae* and *Xanthomonadales Incertae Sedis* (Supplementary Figure 1). In the SS itself, *Cytophagaceae* was the most abundant family, followed by *Nitrosomonadaceae*, *Xanthomonadales Incertae Sedis*, *Chitinophagaceae*, and *Xanthomonadaceae*.

### Antibiotic Resistance in SS-Amended vs. Unamended Soils

Out of the 85 studied ARGs, 77 were amplified by HT-qPCR in both SS-amended and unamended soil (74 ARGs were amplified in the SS itself). In addition, the 10 targeted MGE-genes were amplified by HT-qPCR in the SS itself and SS-amended/unamended soil samples.

**FIGURE 1:**
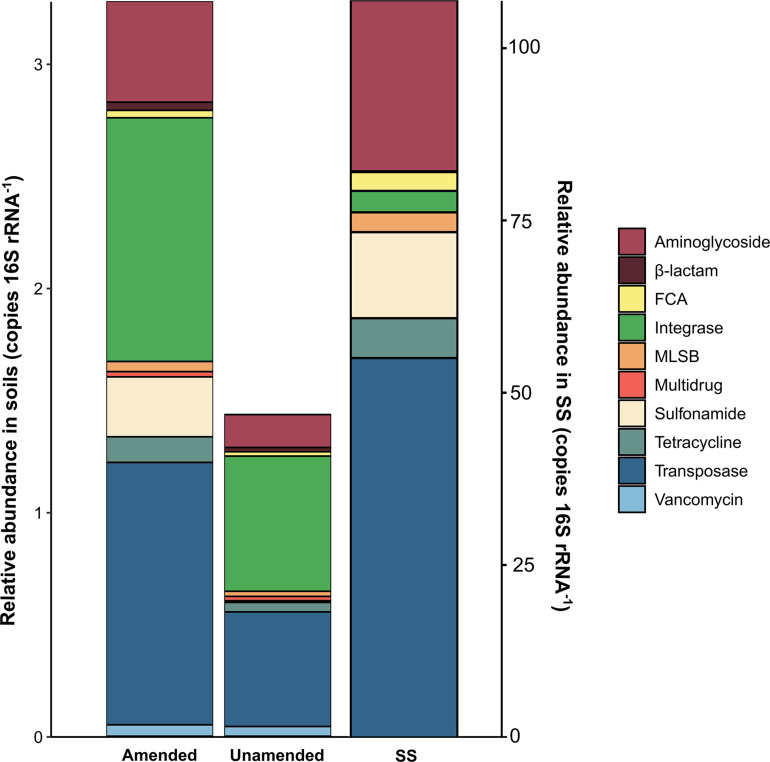
Relative abundances of ARGs and MGE-genes in amended soils, unamended soils and SS.

The relative abundances of ARGs and MGE-genes in amended soils, unamended soils and SS, grouped by antibiotic family or type of MGE-genes, are shown in Figure 1. The relative abundances of ARGs and MGE-genes were higher in SS-amended soils, compared to unamended ones. In particular, the relative abundances of genes conferring resistance to β-lactams (*p* < 0.001), aminoglycosides and transposases (*p* < 0.01), integrase and FCA (*p* < 0.05) were significantly higher in SS-amended vs. unamended soils. Gene relative abundances in the SS itself were several tens of times higher than in soil samples. In SS, transposase genes showed the highest abundance values, followed by aminoglycoside genes.

**FIGURE 2:**
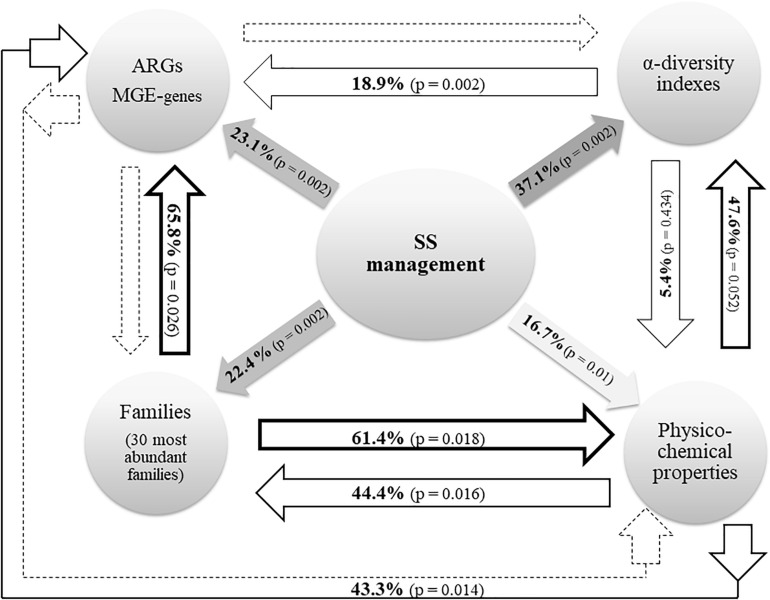
Radial diagram from RDA data. Solid lines represent a significant (*p* < 0.05) effect of the corresponding variable. Dotted lines represent the lack of significant effect. The variable “SS management” includes the dosage of application, the elapsed time after the last application, and the presence/absence of SS. ARGs and MGE-genes: relative abundance (relative to the 16S rRNA gene) of ARGs and MGE-genes. α-diversity: richness, Shannon’s diversity and Simpson’s diversity. Physicochemical properties: OM, pH, CEC, EC, NO3^–^, NH4^+^, total N, Olsen P, K^+^, texture, and pseudo-total metal concentrations (Cd, Cr, Cu, Ni, Pb, and Zn).

The effects of SS management (here, the term “SS management” includes the following three variables: dosage of application, elapsed time after the last application, and presence/absence of SS) on (i) soil physicochemical properties; (ii) the relative abundance of ARGs and MGE-genes; and (iii) the composition (at family rank) and α-diversity of soil prokaryotic communities, along with the interactions between these parameters, are summarized in Figure 2.

**FIGURE 3:**
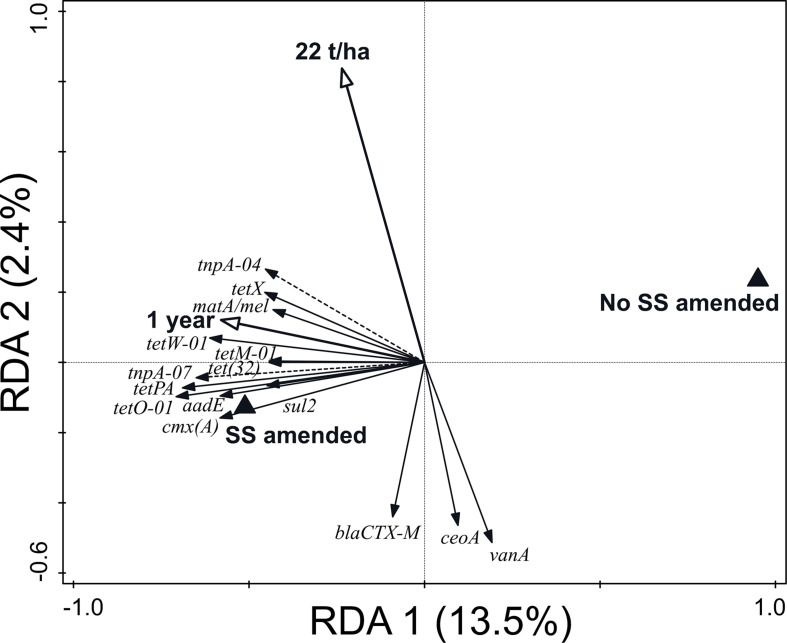
Biplot of the RDA performed with SS management (i.e., dosage of application, elapsed time after the last application, and presence/absence of SS) as explanatory variables, ARGs and MGE-genes relative abundances as response variables, and field and current crop as covariates. Only statistically significant explanatory variables and response variables with the best fit are shown. The explanatory variables explained 23.1% of the variation in ARG and MGE-gene relative abundances.

Regarding the variation of ARG and MGE-gene relative abundances, the RDA represented in Figure 3 (23.1% variation explained, pseudo-F = 2.6, *p* = 0.002) shows that the presence of SS, an elapsed time of 1 year after the last application, and a dosage of application of 22 t SS ha^–1^ were the key factors explaining the distribution pattern of ARG and MGE-gene relative abundances in the studied soils. The factor *presence/absence of SS* was separated along RDA 1. The presence of SS was associated with increased ARG and MGE-gene relative abundances (Figure 3). Pertaining to soil physicochemical properties, 16.7% of the variation of the observed values was explained by SS management (pseudo-F = 1.7, *p* = 0.01). In addition, SS management significantly influenced the distribution of the 30 most abundant prokaryotic families (22.4% variation explained, pseudo-F = 2.5, *p* = 0.002). Finally, 37.1% of the explained variation (pseudo-F = 4.9, *p* = 0.002) in α-diversity values (R, H’, and D indices) was attributable to SS management.

The prokaryotic community composition, according to the 30 most abundant families, was significantly influenced by the physicochemical properties of the studied soils (44.4% variation explained, *p* = 0.016). On the other hand, the 30 most abundant families significantly (*p* = 0.018) explained 61.4% of the variation of soil physicochemical properties. Furthermore, 47.6% of the variation shown by the values of prokaryotic α-diversity (R, H’, and D indices) was due to the physicochemical properties of the studied soils (nonetheless, this influence was not significant, *p* = 0.052). In turn, the distribution pattern of the values of soil physicochemical properties (5.4% variation explained, *p* = 0.434) was also influenced by soil prokaryotic α-diversity.

**FIGURE 4:**
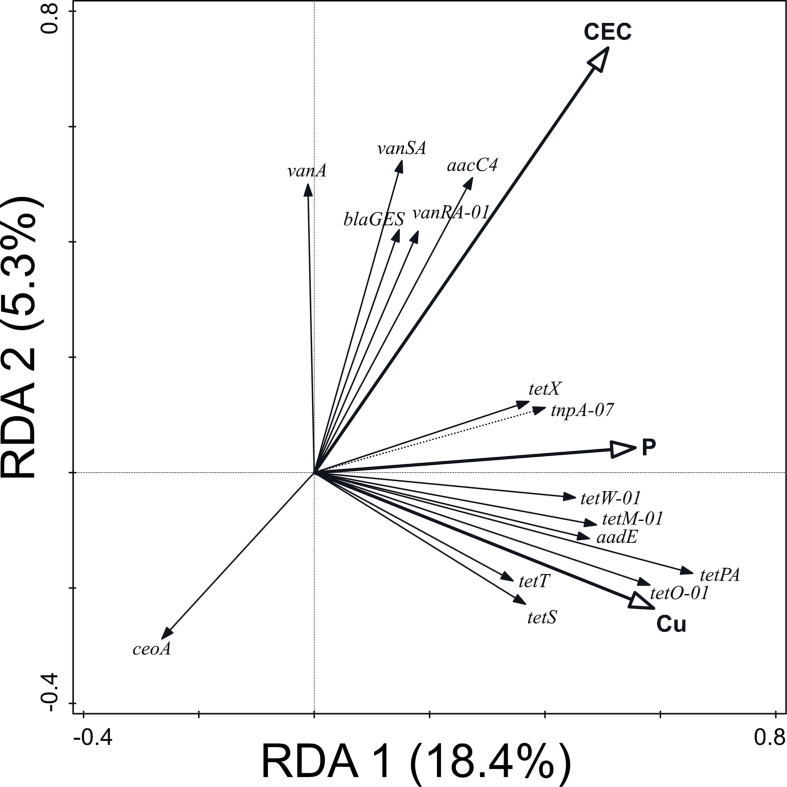
Biplot of the RDA performed with soil physicochemical properties (explaining 43.3% of the variation in ARG and MGE-gene relative abundances) as explanatory variables, the relative abundance of ARGs and MGE-genes as response variables, and field and current crop as covariates. Only statistically significant explanatory variables and response variables with the best fit are shown.

Similarly, 43.3% of the variation in ARG and MGE-gene relative abundances was explained by the values of soil physicochemical properties (pseudo-F = 1.5, *p* = 0.014) (Figure 4). Primarily, this variation was attributable to the pseudo-total Cu concentration (17.1%) but also to the values of CEC and Olsen P. These physicochemical parameters were positively correlated with the relative abundance of most of the ARGs and MGE-genes studied here: pseudo-total Cu concentration and Olsen P were especially (positively) correlated with the abundance of tetracycline resistance genes. Values of CEC were mainly positively correlated with vancomycin resistance genes.

**FIGURE 5:**
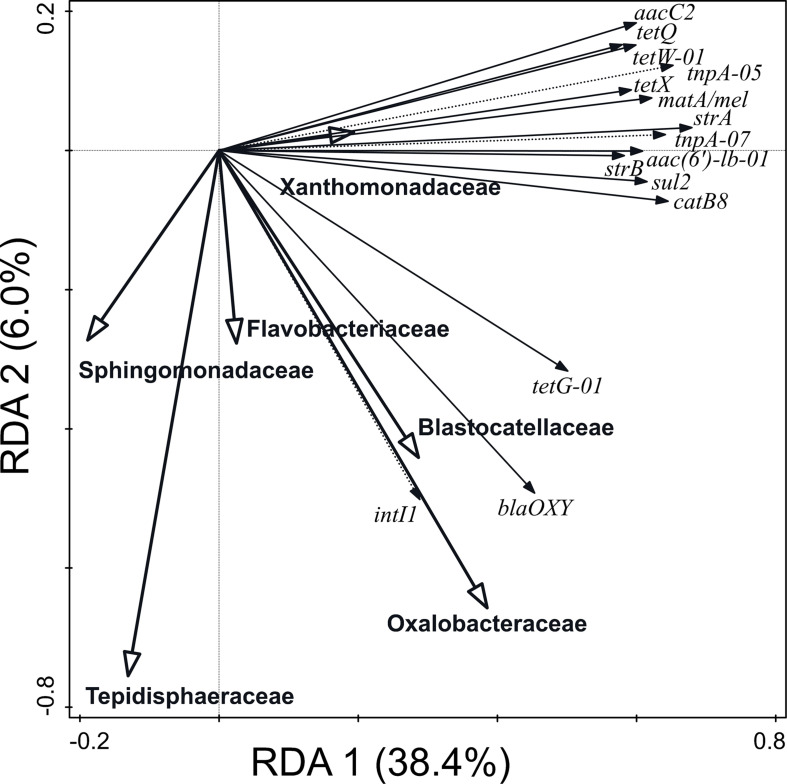
Biplot of the RDA performed with the 30 most abundant families (explaining 65.8% of the variation in ARG and MGE-gene abundances) as explanatory variables, the relative abundance of ARGs and MGE-genes as response variables, and field and current crop as covariates. Only statistically significant explanatory variables and response variables with the best fit are shown.

Furthermore, 65.8% of the variation in ARG and MGE-gene relative abundances was explained by the distribution of the 30 most abundant prokaryotic families (Figure 5). The prokaryotic families which contributed significantly were *Oxalobacteraceae*, *Sphingomonadaceae*, *Xanthomonadaceae*, *Flavobacteriaceae*, *Tepidisphaeraceae*, and *Blastocatellaceae*. The abundance of *Blastocatellaceae*, *Oxalobacteraceae*, and *Flavobacteriaceae* showed positive correlation with the relative abundance of blaOXY (gene conferring resistance to β-lactam antibiotics), tetG-01 (gene conferring resistance to tetracyclines) and intl1 (class 1 integron integrase gene) genes. Particularly, the *Xanthomonadaceae* family positively explained the distribution of ARGs and MGE-genes.

**FIGURE 6:**
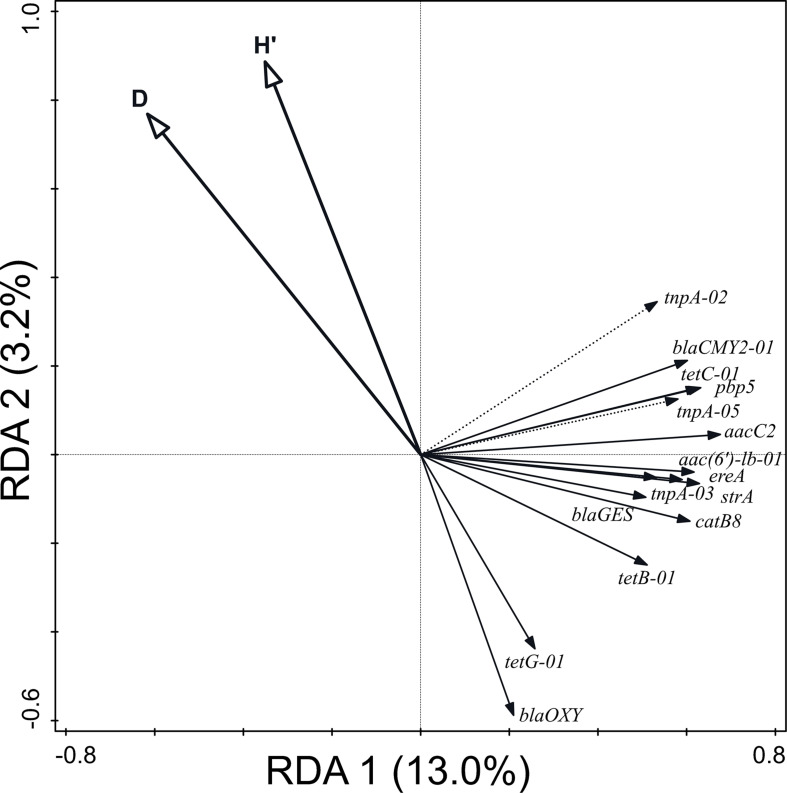
Biplot of the RDA performed with α-diversity indices (explaining 18.9% of the variation in the abundance of ARGs and MGE-genes) as explanatory variables, the relative abundance of ARGs and MGE-genes as response variables, and field and current crop as covariates. Only statistically significant explanatory variables and response variables with the best fit are shown.

Moreover, 18.9% of the variation in the relative abundance of ARG and MGE-genes (*p* = 0.002) was attributable to prokaryotic α-diversity (Figure 6). The values of the Shannon’s and Simpson’s index were negatively correlated with ARG and MGE-gene relative abundances. No correlation was found between richness and the distribution of ARGs or MGE-genes.

**FIGURE 7:**
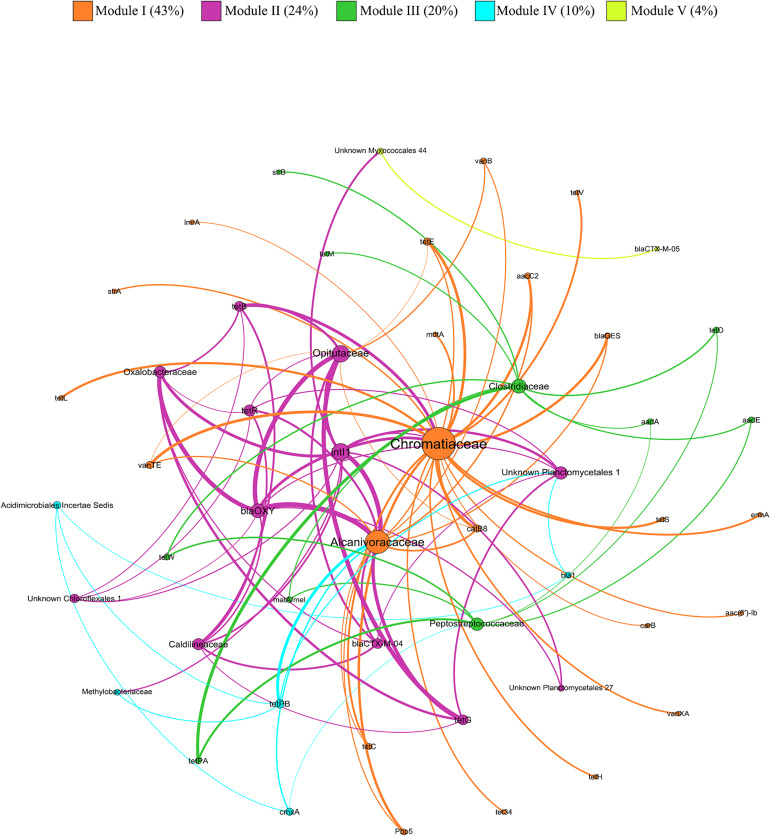
Network analysis of ARGs, MGE-genes (relative abundances) and 12 multi-resistant bacterial families based on Kendall’s correlations. Node size is proportional to the number of connections (degree). An edge represents a significant correlation, where the edge thickness is proportional to Kendall’s correlation coefficient (weight).

Out of the 355 families that showed correlation with the relative abundance of at least one ARG or MGE-gene, 30 showed correlation with at least two genes that are known to confer resistance against several antibiotics. Among these 30 multi-resistant families, 12 showed significantly higher relative abundance values in SS-amended vs. unamended soils (Supplementary Table 1). The correlations between these 12 multi-resistant families and ARGs and MGE-genes were explored by network analysis (Figure 7). The modularity index was 0.465, suggesting that the network had a modular structure (Fortunato and Barthélemy, 2007). The network was divided into five modules (i.e., clusters of nodes that interact more among themselves than with other nodes, compared with a random association). Regarding ARGs and MGE-genes, *intI1* and *blaOXY* had the greatest diversity in terms of possible multi-resistant hosts, with 10 and 8 families, respectively. Furthermore, *Chromatiaceae*, *Alcanivoracaceae*, and *Opitutaceae* presented the highest diversity of ARGs and MGE-genes, with 22, 15, and 10 genes, respectively.

**FIGURE 8:**
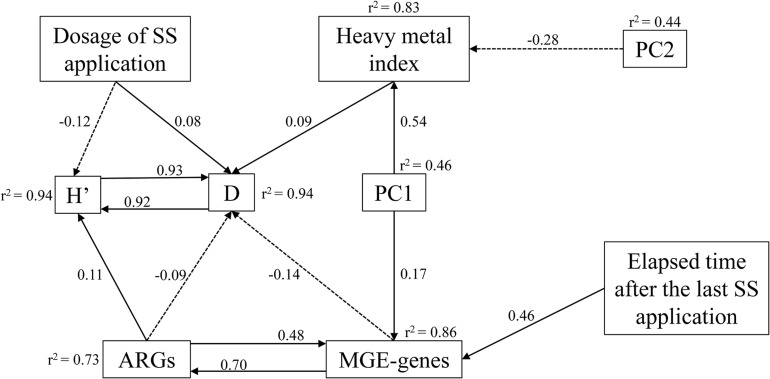
Structural equation models showing direct and indirect effects of prokaryotic diversity indices, dosage of SS application, elapsed time after the last application, HM pollution index, PC1 and PC2 on ARGs and MGE-genes. Arrows indicate positive and negative relationships by solid and dashed lines, respectively. Numbers next to arrows represent standardized estimated regression weights (*p* < 0.05). The *r*^2^-values indicate the proportion of variance explained for each variable. χ^2^ = 31.5, AIC = 133, *P* = 0.416, *df* = 34.

Since many studied variables showed correlation among themselves, a SEM analysis was performed to outline the direct or indirect influence of biotic and abiotic factors on the distribution of ARGs and MGE-genes (Figure 8). The HM pollution index was directly affected by many soil physicochemical properties, such as nutrient contents, OM, clay content, CEC, and EC. The dosage of SS application had a negative and positive influence on the Shannon’s and Simpson’s index, respectively. Specifically, the Simpson’s index (*r*^2^ = 0.94) was directly affected negatively by ARGs and MGE-genes, unlike the HM pollution index and the Shannon’s index. Regarding the standardized regression weights, MGE-genes were primarily regulated by ARGs, followed by the elapsed time after the last application and the PC1 (*r*^2^ = 0.86).

## Discussion

The application of organic amendments to agricultural soil is a common practice because of its potential positive effects on soil quality (Epelde et al., 2018; Urra et al., 2019b). Furthermore, this practice reduces the need for energetically costly synthetic fertilizers, while reusing a material that would otherwise be treated as a waste. Relevantly, in the current scenario of climate change, the application of SS to agricultural soils can help increase their soil OM content (a pertinent objective for many agricultural soils with low OM content), thus enhancing their capacity to sequester carbon. Regrettably, the application of organic amendments to agricultural soil, such as SS, can likewise lead to potential risks for human and environmental health, in particular, due to the presence of traditional and emerging contaminants (Singh and Agrawal, 2007; Alvarenga et al., 2015; Martín et al., 2015). In particular, nowadays, emerging contaminants (e.g., pesticides, pharmaceuticals, nanomaterials, personal and house care products, etc.) are a matter of much concern (Epelde et al., 2018). The possible dissemination of ARGs is of special concern owing to the risk of transfer of ARGs to human pathogens (Marshall and Levy, 2011; Epelde et al., 2018).

In this study, SS-amended soils presented higher Olsen P contents than unamended soils, as previously described (Börjesson and Kätterer, 2018; Urra et al., 2019a). The rate and amount of SS application to agricultural soil is frequently based on the N requirement of crops. Then, other nutrients, such as P and K^+^, can be over-applied (Antoniadis et al., 2015). It is a well-known fact that SS from WWTPs can present elevated levels of P (Alvarenga et al., 2015), a fact that must be taken into consideration as this key nutrient is being depleted at an alarming rate due chiefly to its use in fertilizers. In accordance with other studies (Kirchmann et al., 2017; Bogusz and Oleszczuk, 2018; Urra et al., 2019a), in our study, the application of SS also resulted in significantly higher soil Zn concentrations. Sewage sludge application to agricultural soil can lead to accumulation of potentially toxic metals, with concomitant adverse effects for crop quality (Muchuweti et al., 2006). On the other hand, in accordance with previous studies (Lloret et al., 2016; Urra et al., 2019a), the application of SS resulted in reduced soil microbial diversity, with potential negative consequence for soil functioning and resilience (Delgado-Baquerizo et al., 2016). On the contrary, some authors found an increase in diversity in SS-amended soils (Chen et al., 2016; Bai et al., 2019).

The application of SS to soil can result in an increase of microbial biomass and activity, associated with the supply of easily available carbon sources for the soil microbial communities (García-Gil et al., 2004; Sánchez-Monedero et al., 2004; Fernández et al., 2009). The application of organic amendments has been frequently reported to enhance soil microbial activity and biomass (Dinesh et al., 2010; Zhen et al., 2014; Hernández et al., 2016; Reardon and Wuest, 2016; Das et al., 2017; Urra et al., 2019a). In our study, unexpectedly, no differences in microbial biomass and activity values were found between SS-amended and unamended soils. At soil sampling times, our agricultural fields were planted with different crops (barley, beans, rapeseed, wheat). Taking into account that our soil samples were collected from the topsoil (0–10 cm depth), it is possible that the expected stimulation of soil microbial biomass and activity, derived from the rhizodeposition of readily available carbon sources, might have veiled the positive effect of SS on soil microbial biomass and activity. It is also feasible that, at sampling times, most of the easily available C substrates initially present in SS were long gone (elapsed times after the last SS application range from 1 to 4 years).

In our study, all the sampled soils and the SS itself met the EU Directive 86/278/EEC (European Commision, 1986) regarding total metal concentration values. However, the bioavailable fraction of soil metals is considered more relevant from an environmental risk point of view (Olaniran et al., 2013). Bioavailable metal concentrations in soil usually show an inverse relationship with soil pH (Rieuwerts et al., 1998). The agricultural soils sampled for this study have an alkaline pH (mean value: 8.4 ± 0.1), which could explain the fact that bioavailable metal levels were below the quantification limit, suggesting a lack of metal-induced ecotoxicity. Similarly, Urra et al. (2019a) found that the application of SS increased the total concentration of Cu and Zn in amended soils, but without affecting their bioavailability, possibly due to the high values of soil pH and OM content.

It is a well-known fact that metals might promote the spread of antibiotic resistance via co-selection mechanisms. In consequence, due to the current growing concern over the emergence and dissemination of antibiotic resistance, an increasing interest exists in the study of the generalities and particulars related to the role of metal contamination as selective agent in the proliferation of environmental antibiotic resistance. This co-selection depends on a variety of factors, including the nature and concentration of metal contaminants, as well as the type of antibiotic and its mechanism of action. In any case, the co-selection of antibiotic and metal resistance rests on two main mechanisms: (i) co-resistance, when different resistance determinants are present in the same genetic element; and (ii) cross-resistance, when the same genetic determinant is responsible for resistance to both antibiotics and metals (Baker-Austin et al., 2006). Due to the magnitude of the problem of metal contamination, as well as to the exceptionally long residence times of metals in the environment, metals represent a widespread and recalcitrant selection pressure contributing to the emergence, maintenance and spread of antibiotic resistance factors. In our study, total Cu concentration in soil was positively correlated with the relative abundance of both ARGs and MGE-genes. Other authors have reported that the selective pressure caused by the presence of metals in SS can result in increased abundance of ARGs (Gullberg et al., 2014). In a previous study on the long-term impact of SS on agricultural soil (Urra et al., 2019a), we found a positive correlation between soil Cu and Zn concentrations and the abundance of ARGs and MGE-genes in soils amended with thermally-dried anaerobically-digested SS. Similarly, we observed that all metals were positively correlated with, at least, one ARG (soil metal concentrations also showed positive correlations with MGE-genes, particularly with the gene *tnpA-07*) (Urra et al., 2018).

The content of Olsen P explained a considerable percentage of the variation in the relative abundances of ARGs and MGE-genes in our soils, which could possibly be related to the abovementioned high P content of SS (Daneshgar et al., 2018). Soil CEC was also positively correlated with the relative abundance of ARGs, which could be, at least partly, due to the interaction (e.g., sorption mechanisms) between antibiotics and soil surfaces in SS-amended soils (Tolls, 2001). The distribution and fate of antibiotics in soil hinge on their sorption coefficient value (K_d_) which, in turn, depends on a variety of soil properties, such as soil pH, OM, and CEC (Lützhøft et al., 2000; Chee-Sanford et al., 2009; Wegst-Uhrich et al., 2014). In any event, although environmental parameters such as soil pH and OM content are the most significant variables that affect antibiotic sorption in soil, it is important to take into consideration the concentrations used, the analytical method employed, and the transformations that can occur when determining K_d_ values (Wegst-Uhrich et al., 2014). Antibiotics with low K_d_ values are highly mobile (they are not strongly bound to soil particles) and, hence, more bioavailable and degradable. The principal route for antibiotic degradation in soil is aerobic biodegradation. Antibiotic degradation rates, usually expressed as half-lives, can range from days to years (Boxall et al., 2004). Macrolides and β-lactams have been reported to be less persistent in soil than tetracyclines and quinolones (Boxall et al., 2004).

As stated above, the application of organic wastes of anthropogenic origin, such as SS, can increase the risk of dissemination of ARGs into agricultural soil (Munir and Xagoraraki, 2011). Values of ARG relative abundance in our SS were higher compared to those observed by other authors: in anaerobically digested sludge from South Korea, Yoo et al. (2020) reported relative abundance values of 8.93 × 10^–2^, 5.74 × 10^–2^, and 5.71 × 10^–2^ for MLSB, sulfonamide and tetracycline genes, respectively. Xu et al. (2020) found ARG relative abundance values of 3.3–5.1 × 10^–1^ in SS samples from two Chinese WWTPs operated via anaerobic/anoxic/oxic and anoxic zone nitrification techniques. Han and Yoo (2020) reported ARG relative abundance values from 5.80 × 10^–5^ to 1.20 × 10^–1^ and from 4.62 × 10^–6^ to 4.48 × 10^–1^ in activated and dewatered sludge, respectively. Differences in antibiotic consumption patterns, as well as in SS composition, treatment and management, are possibly responsible for the observed differences in ARG relative abundance values. In any case, it is strongly recommended that SS is properly treated prior to its application in order to minimize or prevent the spread of ARB and ARGs from WWTPs to agricultural soils. This same recommendation was reported by Urra et al. (2019c) in their study on the impact of six different manure-derived amendments on agricultural soil quality. According to Sun et al. (2016), anaerobic digestion generally reduces integron abundance, eliminating the aerobic hosts of such integrons. The presence of the class 1 integron has been reported to be associated with the proliferation of ARGs in soil (Binh et al., 2008). In fact, the *intl1* gene was proposed as a proxy for anthropogenic contamination, due to its relationship with ARGs, disinfectants and metals (Gillings et al., 2015).

In any event, when interpreting data on antibiotic resistance in soil, it must not be forgotten that natural soil microbial communities are potentially a large environmental reservoir of antibiotic resistance (Allen et al., 2009). Although, in this study, thermally-dried and anaerobically-digested SS was used as amendment, out of the 85 ARGs measured here, 77 and 74 were detected in soil samples and SS itself, respectively. Furthermore, the 10 MGE-genes determined in our study were detected in both soil samples and SS.

In our study, the relative abundance of both ARGs and MGE-genes was higher in SS-amended soils, compared to non-amended soils, particularly in those with a more recent SS application, suggesting that the application of thermally-dried anaerobically-digested SS to agricultural soil can increase the risk of environmental antibiotic resistance, as previously described by Urra et al. (2019a). Rahube et al. (2014) reported the impact of fertilizing with anaerobically-digested vs. raw SS on the abundance of antibiotic-resistant coliforms, antibiotic resistance genes, and pathogenic bacteria in soil and on vegetables at harvest. Instead, Rutgersson et al. (2020) did not find evidence of antibiotic accumulation or enrichment of ARGs or ARB in soil amended with digested and stored SS at doses of up to 12 tons per hectare every 4 years.

The use (above all, the overuse and misuse) of antibiotics for animal, human and agricultural purposes has led to the emergence and dissemination, by HGT, of antibiotic resistance among bacteria. The spread of antibiotic resistance by HGT is an ancient phenomenon. Nonetheless, the use of antibiotics has raised the corresponding selective pressure, thus increasing HGT (von Wintersdorff et al., 2016). As abovementioned, our results showed a stimulatory effect of SS on the relative abundance of both ARGs and MGE-genes, especially when the elapsed time after the last application was only 1 year, in agreement with previous studies (Chen et al., 2016; Xie et al., 2016). *A priori*, the increased relative abundances of ARGs and MGE-genes in SS-amended soils could be the consequence of: (i) an increase microbial growth induced by the input of organic carbon sources present in the SS itself; and/or (ii) the dissemination of ARGs from SS to soil microorganisms through HGT. Since the intensity of these processes will decrease over time after the application of SS, it is expected that the resistome risk will also decrease over time after the last application of SS. In a much shorter microcosm experiment, Chen C. et al. (2019) found that risk scores for amended soils decreased to levels comparable to those of unamended soil after just 120 days. However, as stated before, the persistent accumulation of heavy metals in SS-amended soils provide a long-term selective pressure which can result in co-selection for antibiotic resistance (Song et al., 2017) and, thus, a persistent resistome risk.

Urra et al. (2019c) observed that genes encoding MGEs (*tnpA*, *intI1*) were positively correlated with ARGs, suggesting a risk of dissemination of antibiotic resistance via HGT in agricultural soils, as a result of the application of livestock manure-derived amendments. Garbisu et al. (2018) screened metal contaminated soil treated with organic wastes for the presence of MGEs, confirming the occurrence of conjugative IncP-1 and mobilizable IncQ plasmids, as well as of class 1 integrons, suggesting that bacteria from those soils had gene-mobilizing capacity with implications for the dissemination of resistance factors. Moreover, their data pointed out the role of spontaneous mutations in achieving low-level antibiotic resistance in a short time, which was associated with a trade-off in the capability to metabolize specific carbon sources (Garbisu et al., 2018).

Regarding prokaryotic taxa at phylum level, the two dominant phyla in SS were *Proteobacteria* and *Bacteroidetes*. Both phyla have previously been reported as dominant taxa in SS (Liao et al., 2018; Zhao et al., 2019) and can harbor ARGs (Sun et al., 2016). In accordance with previous studies (Zhang et al., 2019), in our soils, *Xanthomonadaceae* was linked with the relative abundance of ARGs and MGE-genes. Interestingly, this family was the fifth most abundant family in the SS itself. In their study on the long-term impact of SS application of agricultural soil, Urra et al. (2018) found that some ARGs correlated positively with particular prokaryotic taxa, being Gemmatimonadetes the taxon with the greatest number of positive correlations at phylum level. However, no positive correlation was detected between prokaryotic taxa and genes encoding resistance to sulfonamides and FCA (Urra et al., 2018).

Interestingly, Shannon’s and Simpson’s index values correlated negatively with ARG and MGE-gene relative abundances, as previously reported (Chen Q. L. et al., 2019), suggesting that a decrease in prokaryotic diversity could facilitate the proliferation and spread of ARGs and MGE-genes in soil. Since microbial diversity is essential for ecosystem functioning and resilience (Delgado-Baquerizo et al., 2016), the impact of organic amendments on such diversity must be carefully studied.

According to data from our Kendall’s correlation analysis, 30 families showed correlation with at least two genes that are known to confer resistance against several antibiotic families. Among these 30 multi-resistant families, 12 showed a higher abundance in SS-amended vs. unamended soils, pointing out to an increased risk of antibiotic resistance.

The network analysis is a powerful tool to identify key ARGs (with potential as indicators or proxies) and to delineate potential ARGs hosts (Chen et al., 2016). In our network analysis, the families *Chromatiaceae*, *Alcanivoracaceae*, and *Opitutaceae* were identified as the most common potential hosts of ARGs and MGE-genes in SS-amended soils. Many species belonging to *Chromatiaceae* are photosynthetic sulfur bacteria which contribute to water purification in sewage lagoons (Holm and Vennes, 1970). The family *Alcanivoracaceae* contains bacterial species that harbor Type IV and Type II secretion system genes (Yakimov et al., 2019). The Type II pathway is associated with organisms that form biofilms (Sandkvist, 2001). Bacteria within biofilms can be 10–1,000 times more resistant to antibiotics, compared to corresponding planktonic cells (Ceri et al., 1999). In accordance with Chen Q. L. et al. (2019), the family *Opitutaceae* was strongly correlated with a tetracycline resistance gene (tetG). The higher relative abundance shown by these three families in SS-amended soils, together with their positive correlation with the abundance of ARGs and MGE-genes, suggests their potentially important role in AR in SS-amended agricultural soils.

Structural equation models facilitate the study of complex interactions. In our study, soil physicochemical parameters (OM, CEC, EC, clay content, pH, soil nutrients) were major predictors of the HM pollution index. According to our SEM analysis, the composition and relative abundance of ARGs were mainly driven by MGE-genes, pointing out to the well-known role of HGT for the observed resistome (Smillie et al., 2011). The elapsed time after the last application contributed to the observed variation in MGE-genes diversity and abundance, suggesting that the acquisition of genetic material from non-parental lineages by HGT is dependent on such elapsed time. Our SEM confirmed the recognized crucial role of MGEs in shaping the patterns of ARGs and facilitating their dissemination. Indeed, in this study, MGE-genes (which were themselves affected by parameters such as soil OM and nutrient contents, the elapsed time after the last application of SS, and the distribution of ARGs) have shown their critical role for AR dissemination in agricultural soils.

## Conclusion

In our SS-amended soils, the composition of prokaryotic communities was mainly influenced by the soil’s physicochemical properties. The 30 most abundant families within the soil prokaryotic community accounted for a considerable percentage (66%) of the total variation of ARG and MGE-gene relative abundances. Soil prokaryotic α-diversity was negatively correlated with the relative abundance of ARGs and MGE-genes, suggesting that a decrease in soil prokaryotic diversity could facilitate the proliferation and spread of ARGs and MGE-genes in soil. The families *Chromatiaceae*, *Alcanivoracaceae*, and *Opitutaceae* were identified as the most common potential hosts of multi-resistant genes in SS-amended soils. According to the SEM performed here, the diversity and abundance of MGE-genes are critical for AR dissemination in agricultural soils. We concluded that agricultural soils amended with our thermally-dried anaerobically-digested SS showed increased risk of antibiotic resistance dissemination. Much research and awareness is needed for (i) a safe reduction of antibiotic consumption and (ii) the development of innovative treatments for SS (prior to its application) in order to reduce the risk of emergence and spread of ARGs and MGE-genes in SS-amended agricultural soils and then crops. Until then, SS should be applied conservatively in agricultural soils.

## Data Availability Statement

The datasets presented in the study are publicly available. The sequencing data can be found here: https://www.ebi.ac.uk/ena, PRJEB40822. The HT-qPCR datased is included in Supplementary Table 2.

## Author Contributions

CG, IA, and LE designed the study. LJ and LE performed the analytical work and analyzed data. LJ, CG, IA, and LE wrote the manuscript. All authors revised the final version of the manuscript.

## Conflict of Interest

The authors declare that the research was conducted in the absence of any commercial or financial relationships that could be construed as a potential conflict of interest.
