# Supplementary material for: Agricultural Soils Amended With Thermally-Dried Anaerobically-Digested Sewage Sludge Showed Increased Risk of Antibiotic Resistance Dissemination
Source: Front Microbiol. 2021 Apr 28;12:666854. doi: 10.3389/fmicb.2021.666854 (PMC8113772; doi:10.3389/fmicb.2021.666854)
Supplement: Supplementary file 1 [file Data_Sheet_1.DOCX]

Supplementary Material

**Supplementary Table 1.** Relative abundance of multi-resistant bacterial families in SS-amended and unamended soils. *: p<0.05; **: p<0.01; ***; p<0.001.

| **Family** | **Amended** | **Unamended** |
| --- | --- | --- |
| Acidimicrobiales Incertae Sedis ** | 1.43E-04 | 9.75E-05 |
| Alcanivoracaceae ** | 1.65E-04 | 3.05E-05 |
| Caldilineaceae ** | 1.71E-03 | 1.29E-03 |
| Chromatiaceae | 1.39E-05 | 1.21E-05 |
| Chthoniobacteraceae | 1.05E-02 | 9.29E-03 |
| Clostridiaceae ** | 5.15E-04 | 3.18E-04 |
| Methylobacteriaceae * | 5.94E-03 | 4.86E-03 |
| Oceanospirillaceae | 1.82E-03 | 1.63E-03 |
| Opitutaceae * | 6.52E-03 | 5.42E-03 |
| Oxalobacteraceae ** | 2.17E-02 | 1.53E-02 |
| Peptostreptococcaceae ** | 5.00E-04 | 2.60E-04 |
| Rhizobiaceae | 2.09E-03 | 2.01E-03 |
| Tepidisphaeraceae | 1.94E-02 | 1.62E-02 |
| Unknown Bdellovibrionales family 1 | 3.67E-05 | 2.72E-05 |
| Unknown Bdellovibrionales family 29 | 4.22E-06 | 5.42E-06 |
| Unknown Chlamydiales family 2 | 1.38E-05 | 1.93E-05 |
| Unknown Chloroflexales family 1 * | 1.44E-04 | 9.62E-05 |
| Unknown Myxococcales family 44 *** | 5.74E-06 | 1.07E-06 |
| Unknown Oligoflexales family 1 | 3.50E-05 | 2.34E-05 |
| Unknown Phycisphaerales family 6 | 2.59E-05 | 1.73E-05 |
| Unknown Planctomycetales family 1 ** | 7.04E-05 | 3.70E-05 |
| Unknown Planctomycetales family 15 | 2.24E-05 | 1.74E-05 |
| Unknown Planctomycetales family 2 | 7.95E-05 | 6.15E-05 |
| Unknown Planctomycetales family 27 * | 1.49E-05 | 6.96E-06 |
| Unknown Planctomycetales family 69 | 6.14E-06 | 6.11E-06 |
| Unknown Tepidisphaerales family 10 | 3.75E-05 | 2.46E-05 |
| Unknown Tepidisphaerales family 4 | 9.16E-05 | 8.98E-05 |
| Verrucomicrobiaceae | 3.21E-03 | 2.88E-03 |
| Vicinamibacter family incertae sedis | 6.22E-04 | 4.83E-04 |
| Xiphinematobacteraceae | 1.17E-03 | 9.94E-04 |

**
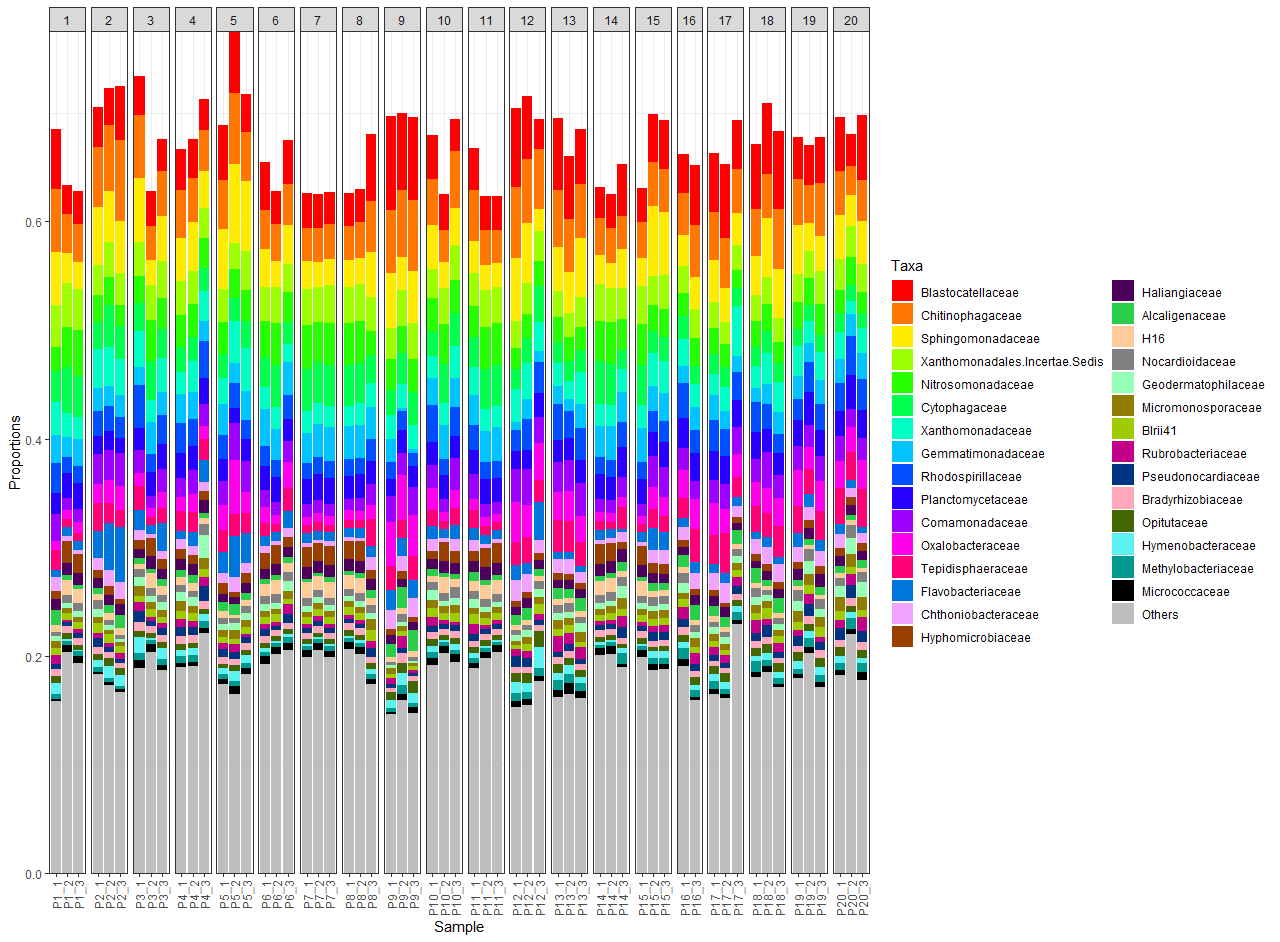
**

**Supplementary Figure 1.** Barplots of the 30 most abundant bacterial taxa at family rank grouped by agricultural field.
